# Association between dietary total antioxidant capacity and the risk of stroke: a nested case-control study

**DOI:** 10.1186/s40795-024-00867-5

**Published:** 2024-04-15

**Authors:** Adrina Habibzadeh, Mehran Rahimlou, Mahdi Ravankhah, Farhad Vahid, Reza Tabrizi

**Affiliations:** 1 Student Research Committee, Fasa University of Medical Sciences, Fasa, Iran; 2 USERN Office, Fasa University of Medical Sciences, Fasa, Iran; 3 Department of Nutrition, School of Public Health, Zanjan University of Medical Sciences, Zanjan, Iran; 4 School of Medicine, Shiraz University of Medical Sciences, Shiraz, Iran; 5 Nutrition & Health Research Group, Department of Precision Health, Luxembourg Institute of Health, Strassen, Luxembourg; 6 Noncommunicable Diseases Research Center, Fasa University of Medical Sciences, Fasa, Iran; 7 Clinical Research Development Unit, Valiasr Hospital, Fasa University of Medical Sciences, Fasa, Iran

**Keywords:** Cerebrovascular disease, Oxidative damage, Dietary antioxidant index

## Abstract

**Background:**

Oxidative stress after ischemic stroke contribute to neuronal cell injury. Unhealthy and unbalanced dietary patterns can increase the risk of several diseases, including stroke and cardiometabolic ones. However, the association between dietary total antioxidant capacity (DTAC) of antioxidant and stroke is controversial. Our study aimed to establish a correlation between DTAC and its impact on the occurrence of stroke.

**Methods:**

This nested case–control study included 79 stroke cases and 158 healthy controls. We used data from the Fasa Adults Cohort Study (FACS) comprising 10,035 individuals at baseline. To assess the nutritional status of each individual, a 125-item food frequency questionnaire (FFQ) has been used to evaluate their dietary habits and intakes over the past year. DTAC was calculated using the ferric-reducing antioxidant power (FRAP) international databases. The stroke was confirmed by an experienced neurologist using standard imaging methods. Conditional logistic regression analyses were performed to evaluate the association between DTAC and stroke.

**Results:**

The assessment of DTAC revealed that there was no statistically significant distinction between cases (mean ± SD: 5.31 ± 2.65) and controls (5.16 ± 2.80) with a p-value of 0.95. Even after adjusting for the potentially important confounding factors such as age, sex, event time, energy intake, smoking, hypertension, and diabetes, the association remains non-significant (adjusted odds ratio (OR) = 1.06, 95% CI: 0.94, 1.20, p-value = 0.33).

**Conclusions:**

Our results did not confirm a significant link between DTAC and stroke risk. These findings emphasize the intricate interplay of factors influencing stroke risk and highlight the need for further research to unravel these relationships more comprehensively.

## Introduction

Stroke is a global public health concern, with major effects on survivors’ quality of life [1]. Although the incidence of stroke has decreased in some Western countries, it has increased in developing countries, notably those in the Middle East, in recent decades [2]. While the precise prevalence of stroke in Iran is unknown, a systematic review suggests that 40 people out of every 100,000 have this disease [3]. As a result, primary stroke prevention has emerged as a critical public health priority, particularly in developing countries [4].

Hypertension, diabetes, dyslipidemia, cardiovascular disease, obesity, physical inactivity, and smoking are all well-known stroke risk factors [5–10]. Lifestyle factors, including eating habits, have been identified as key contributors to stroke prevention or development [5, 11, 12]. Diets with high amounts of saturated or trans fatty acids, as well as fried foods, are associated with increased stroke risk [13–15]. On the other hand, it has been shown that following diets with high amounts of fruit and vegetable consumption acts as a protective factor against the risk of stroke [16].

Adhering to an active lifestyle and a healthy diet is one of the most important preventive measures against stroke [17]. Apart from certain foods like fruits, vegetables, grains, and olive oil, eating habits that are centered on a plant-based diet have been demonstrated to provide protection against stroke. Examples of such diets include the Mediterranean diet and the Dietary Approaches to Stop Hypertension (DASH) diet [18, 19]. Also, there is a correlation between the intake of antioxidant and the risk of stroke, as indicated by several studies [20–22]. Oxidative stress, caused by an imbalance between the production of reactive oxygen species (ROS) and the body’s antioxidant defense mechanisms, has been implicated in the pathogenesis of stroke [23, 24]. Antioxidants are compounds that scavenge ROS and protect cells from oxidative damage and have been suggested to play a role in reducing the risk of stroke [25, 26]. ROS contribute to ischemic damage via a variety of mechanisms, including inducing lipid peroxidation in the mitochondrial membrane and enhancing polymerization and polysaccharide breakdown [23, 27–29]. Cross-linking and oxidation have a negative impact on macromolecules such as deoxyribonucleic acid (DNA), ribonucleic acid (RNA), polysaccharides, and amino acids, causing them to lose their original activity or function. Because of their high reactivity to oxidized lipids and proteins, ROS disrupt endothelial cells and increase blood-brain barrier (BBB) permeability. They also promote inflammation and an immune response by modulating the expression of pro-inflammatory mediators, exacerbating brain injury. Furthermore, ROS contribute to the hastening of delayed neuronal death [23, 29, 30].

Dietary total antioxidant capacity (DTAC) is a measure of the overall antioxidant capacity of a diet, taking into account the synergistic effects of different antioxidants and their interactions with each other [31, 32]. Evidence from studies, including those conducted among Italian [33] and Iranian populations [22], suggests that higher DTAC levels are associated with a reduced risk of stroke. However, the relationship between DTAC and stroke risk remains a subject of debate. Conflicting findings from multiple studies across different populations have contributed to the controversy [34–36]. Furthermore, some studies found no significant association between DTAC and stroke risk in various age groups [35].

Therefore, the association between DTAC and stroke remains controversial. The objective of this study was to examine the correlation between DTAC and the risk of stroke in the Iranian population, after adjusting for potential risk factors.

## Materials and methods

### Study population

Data for this nested case-control study were obtained from the Fasa Adult Cohort Study (FACS), a long-term population-based study conducted between November 2014 and June 2019. The FACS consisted of 10,035 individuals aged 35 to 70 without physical or mental disabilities. This cohort study aimed to identify risk factors for noncommunicable diseases in this population [37].

After the initial registration, biological samples were collected from the participants. Subsequently, a comprehensive physical examination was conducted, and various anthropometric measurements were taken. Additionally, detailed information about the participants’ demographics, socioeconomic status, lifestyle habits, dietary patterns, and medical histories was obtained through interviews. We excluded patients with more than 10% missing data from our analysis. Also, we excluded patients with reported calorie intake exceeding 4000 kcal or falling below 600 kcal from our analysis.

The study enrolled seventy-nine patients with acute ischemic stroke, diagnosed based on clinical presentation, neurologic examination, and brain imaging results using either computed tomography (CT) or magnetic resonance imaging (MRI) with diffusion-weighted imaging. The control group comprised 158 healthy individuals matched for age, sex, and event time, with no known history of stroke.

### Dietary consumption assessment

To assess participants’ typical dietary intake, we utilized a validated Food Frequency Questionnaire (FFQ) consisting of 125 items [37]. This questionnaire was created using popular Iranian foods [38]. The validity and reliability of this questionnaire in stroke patients in Iran has already been investigated and confirmed [39, 40]. Several previous studies have been used this FFQ for evaluation of dietary indices [41, 42]. We opted for the completion of the Food Frequency Questionnaire (FFQ) by the patients’ family members due to several factors. Many of the patients involved in the study were uneducated, and a subset had experienced strokes, rendering them unable to effectively respond to the questionnaire. Given these circumstances, we deemed it more practical and reliable to have their family members provide the dietary information on their behalf. This decision was primarily motivated by the common occurrence of impaired memory or cognitive challenges among stroke patients, which could potentially compromise the accuracy of self-reported dietary intake data. By involving family members in the completion of the FFQ, we aimed to ensure a more comprehensive and accurate assessment of the patients’ dietary habits, thereby enhancing the reliability of our study findings. Participants were requested to indicate the frequency and quantity of each food item consumed on a daily, weekly, monthly, and yearly basis over the past year, taking into account standard portion sizes. These reported frequencies were then converted to daily intake values, which were then converted to grams per day using the Nutritionist IV software (version 7.0). Finally, the total nutrient and total energy intakes were calculated by adding the values obtained from each food item.

### Total antioxidant capacity calculation

We calculated the DTAC by referring to previously published studies that provided DTAC values based on the ferric reducing antioxidant power (FRAP) measurements of the 106 specific food items chosen for this study. The FRAP assay measures dietary antioxidants’ ability to convert ferric ions to ferrous ions. These FRAP values are given in millimoles per 100 g of food (mmol/100 g) [43].

When similar food items didn’t exist in Iranian culture (for example, different types of bread), we calculated the overall mean FRAP value for those items. We then multiplied the consumption frequencies of each food item by their corresponding FRAP values and added these products together to calculate the DTAC for each participant.

In our study cohort, physical activity was assessed using the International Physical Activity Questionnaire (IPAQ), a widely recognized instrument endorsed for its validity and reliability by various studies [44]. IPAQ measures physical activities across four domains: activities at work, commuting, housework, and leisure time. This multidimensional approach captures activities ranging from walking to more intense levels of physical exertion.

### Ethics approval and consent to participate

Our research protocol followed the guidelines set forth in the Helsinki Declaration, and it received approval from the Fasa University of Medical Sciences Research Council and Ethics Committee (approval code: IR.FUMS.REC.1401.241). Moreover, the participants in our study provided written consent to participate in the research.

### Statistical analysis

Data were expressed as mean ± standard deviation and categorical variables were described using numbers (percent). To examine whether the variables are normally distributed, we used from the Kolmogorov–Smirnov test. Based on their DTAC, the participants were divided into tertiles. We used the analysis of covariance (ANCOVA) for continuous variables and the Chi-square test for categorical variables to assess differences in general characteristics among DTAC tertiles and between cases and controls. We used conditional logistic regression in different models to investigate the relationship between DTAC and stroke risk after adjusting according to the most important and clinical variables. First after controlling for SBP and DBP, adjustments were made in the second model for adjusted for energy intake, and smoking. In the other model, additional adjustments included hypertension, diabetes, hyperlipidemia, and overweight. P-values less than 0.05 were considered statistically significant. The Statistical Package for Social Sciences (SPSS Corp, version 20, Chicago, IL, USA) was used for all statistical analyses.

## Results

### General characteristics of the study population

Overall, data from 79 cases and 158 controls were analyzed. Table 1 provides a comprehensive overview of the demographic and health-related characteristics of the study population, highlighting differences between cases and controls, as well as providing insights into DTAC levels.

**Table 1:** General characteristics of the study population^a^

Variables	Total (*n* = 237)	Case (*n* = 79)	Control (*n* = 158)	*P*-value^c^
DTAC	5.20 ± 2.74	5.31 ± 2.65	5.16 ± 2.80	0.957
Age (y)	57.85 ± 7.93	57.82 ± 7.99	57.84 ± 7.97	0.986
Weight (kg)	67.38 ± 15.05	70.81 ± 15.19	65.77 ± 14.80	0.641
BMI (kg/m^2^)	25.53 ± 5.08	26.90 ± 4.79	25.12 ± 5.16	0.287
WC (cm)	93.86 ± 12.97	96.54 ± 12.34	92.71 ± 13.10	0.533
Male (%)	135(56.96%)	45 (57.0%)	90 (57%)	0.998
Physical activity (MET-min/day)	4001.18 ± 5290.63	3876.12 ± 5123.43	4352.64 ± 6435.23	0.516
Current smoker (%)	96 (40.50%)	30 (38.0%)	66 (41.8%)	0.674
**Hypertension (%)**	**83(35%)**	**41 (51.9%)**	**42 (26.6%)**	**< 0.001**
**Overweight** ^**b**^ **(%)**	**122 (51.47%)**	**50 (63.3%)**	**72 (45.6%)**	**0.013**
Diabetes (%)	48(20.25%)	21(26.6%)	27(17.1%)	0.122
Hyperlipidemia (%)	60(25.31%)	26 (32.9%)	34 (21.5%)	0.081
**Anticoagulant use (%)** ^**d**^	**30(12.65%)**	**30 (38.0%)**	**0 (0.00%)**	**< 0.001**
**SBP (mmHg)**	**118.7 ± 20.52**	**127.28 ± 22.57**	**114.57 ± 18.33**	**0.023**
DBP (mmHg)	77.33 ± 13.06	81.11 ± 13.55	75.52 ± 12.51	0.691
WBC	6.53 ± 1.63	6.86 ± 1.79	6.39 ± 1.50	0.101
RBC	4.97 ± 0.63	5.04 ± 0.78	4.94 ± 0.53	0.068
Hgb	14.66 ± 1.68	14.88 ± 1.86	14.58 ± 1.56	0.300
HCT	41.92 ± 4.43	42.67 ± 5.02	41.63 ± 4.02	0.161
MCV	85.14 ± 8.05	85.57 ± 8.17	85.01 ± 8.00	0.939
MCH	29.73 ± 3.33	29.86 ± 3.40	29.68 ± 3.31	0.69
Platelets	266.03 ± 67.37	268.87 ± 70.89	264.76 ± 65.92	0.663
**TG**	**134.72 ± 117.33**	**162.12 ± 188.29**	**122.43 ± 60.89**	**0.015**
LDL	112.27 ± 34.69	114.13 ± 40.75	111.44 ± 31.70	0.561
Chol	185.63 ± 48.85	189.74 ± 64.39	183.78 ± 40.08	0.385
**HDL**	**47.20 ± 11.61**	**44.62 ± 12.08**	**48.36 ± 11.23**	**0.021**
BUN	37.20 ± 4.06	14.44 ± 3.47	14.80 ± 4.31	0.529

BMI, Body mass index; BUN, blood urea nitrogen test; Chol, cholesterol; DTAC, Dietary total antioxidant capacity; DBP, diastolic blood pressure; Hgb, hemoglobin; HCT, hematocrit; MCV, mean corpuscular volume; MCH, mean corpuscular hemoglobin; HDL, high density lipoprotein; LDL, low density lipoprotein; RBC, red blood cells; TG, triglyceride

^a^ All data are means ± standard deviations unless indicated ^b^ Overweight was defined as 25 ≥ BMI > 30 kg/m^2^. c, P-value calculated by independent t test

Firstly, when assessing DTAC, there was no statistically significant distinction between cases and controls (*P* = 0.957). Gender distribution showed no significant difference, with 57% males among cases and controls (*p* = 0.998). As measured by metabolic equivalent (MET)-min/day, physical activity levels remained comparable between cases and controls, revealing no statistically significant contrast (*P* = 0.516).

Furthermore, cases had a significantly higher occurrence of obesity, characterized as having a Body Mass Index (BMI) of ≥ 30 kg/m^2^, with a prevalence of 63.3%, in contrast to controls, who had a prevalence of 45.6% (*P* = 0.013). There was a noticeable difference in the use of anticoagulants between cases and controls, as cases had a significantly higher prevalence of 30.0%, while controls had a prevalence of 0.00% (*P* < 0.001). Additionally, systolic blood pressure (SBP) was significantly higher in cases compared to controls (*P* = 0.023).

### Dietary intakes of study participants

Table 2 provides a detailed summary of the dietary intake among study participants.

The results of our study showed that the mean calorie intake in the case and control groups was significantly different (2682.14 ± 949.85 Kcal/day for the case group and 2937.65 ± 918.02 Kcal/day for the control group; *P* = 0.047). In terms of macronutrients and micronutrients intake, protein intake among cases (88.48 ± 32.16 g/day) was slightly lower than that among controls (94.41 ± 32.80 g/day), although this distinction did not reach statistical significance (*P* = 0.188). Upon examination of daily selenium intake, it was discovered that the case group had a mean intake of 123.95 ± 54.95 mg/day, which was significantly lower than the control group’s intake of 140.90 ± 64.21 mg/day (*P* = 0.046).

**Table 2:** Dietary intakes of study participants

Variables	Total (*n* = 237)	Case (*n* = 79)	Control (*n* = 158)	*P* ^a^
**Total energy (kcal/d)**	2833.04 ± 954.46	2682.14 ± 949.85	2937.65 ± 918.02	**0.047**
Proteins (g/day)	92.67 ± 32.65	88.48 ± 32.16	94.41 ± 32.80	0.188
Fats (g/day)	69.27 ± 25.49	65.42 ± 24.60	71.65 ± 25.22	0.072
Carbohydrates (g/day)	433.09 ± 174.57	404.17 ± 160.01	444.64 ± 179.28	0.091
Omega-3 fatty acids (g/d)	0.134 ± 0.05	0.13 ± 0.05	0.14 ± 0.06	0.357
Iron (mg/d)	21.11 ± 7.47	20.49 ± 6.99	21.27 ± 7.62	0.446
**Selenium (mg/d)**	**137.67 ± 64.96**	**123.95 ± 54.95**	**140.90 ± 64.21**	**0.046**
Vitamin C (mg/d)	115.97 ± 50.78	117.10 ± 55.14	114.58 ± 48.39	0.718
Vitamin A (mg/d)	590.23 ± 380.65	604.29 ± 422.60	575.30 ± 345.03	0.573
Vitamin E (mg/d)	7.16 ± 2.30	6.99 ± 2.40	7.29 ± 2.23	0.342

All data is presented as a means ± standard deviations

^a^, calculated by independent t test

### Energy-adjusted DTAC

Table 3 reveals the distinct dietary intake of control groups across tertiles of DTAC. It has been reported that participants in the tertile 3 of DTAC exhibited a significantly higher vitamin C intake (127.01 ± 5.35) compared to those in tertiles 1 and 2 (106.44 ± 5.48 and 113.55 ± 5.37, respectively) (*P* = 0.026). However, we couldn’t find any significant differences among the DTAC tertiles regarding other macro and micronutrients (*P* > 0.05).

**Table 3:** Characteristics of study participants according to tertiles of energy-adjusted dietary total antioxidant capacity ^a^

	Energy-adjusted DTAC			
Variables	Tertile 1 (*n* = 79)	Tertile 2 (*n* = 79)	Tertile 3 (*n* = 79)	*P* ^b^
Total energy (kcal/d)*	2766.90 ± 108.22	2860.84 ± 106.23	2930.77 ± 105.62	0.558
Proteins (g/day)	92.21 ± 1.88	91.61 ± 1.84	94.30 ± 1.84	0.555
Fat (g/day)	69.09 ± 1.87	69.62 ± 1.83	70.50 ± 1.82	0.862
Carbohydrates (g/day)	430.04 ± 13.06	436.88 ± 12.80	430.63 ± 12.75	0.917
Omega-3 fatty acids (g/d)	0.13 ± 0.00	0.14 ± 0.00	0.14 ± 0.00	0.639
Iron (mg/d)	20.11 ± 0.63	21.61 ± 0.61	21.47 ± 0.61	0.163
Selenium (mg/d)	133.35 ± 5.02	135.17 ± 4.92	138.28 ± 4.90	0.777
Vitamin E (mg/d)	6.95 ± 0.26	7.17 ± 0.26	7.46 ± 0.26	0.397
**Vitamin C (mg/d)**	**106.44 ± 5.48**	**113.55 ± 5.37**	**127.01 ± 5.35**	**0.026**
Vitamin A (mg/d)	585.80 ± 43.04	570.58 ± 42.19	587.20 ± 42.00	0.954

Data for other dietary variables are adjusted for age and total energy intake *P* < 0.05 was considered as statistically significant.

*Data for energy intake are adjusted for age

^a^ All data is presented as a means ± standard deviations unless indicated, ^b^ Obtained by the use of ANCOVA

### Association between DTAC and stroke

Table 4 presents the results of the multivariate-adjusted odds ratios (ORs) and 95% confidence intervals (CIs) for assessing the association between DTAC and the risk of stroke, with the control group as the reference category. These analyses were carried out using different models with varying levels of adjustment. In the first model, which is conditioned on the matching factors of age, sex, and event time, the OR for stroke was 1.02 (95% CI: 0.92, 1.13), indicating that there was no statistically significant association between DTAC and the risk of stroke (*p* = 0.68). Also, we couldn’t find any significant correlation between DTAC and stroke in the fully adjusted model (OR = 1.06, 95% CI: 0.94, 1.20; *P* = 0.33).

**Table 4:** Multivariate-adjusted ORs and 95% CIs for stroke in relation to dietary total antioxidant capacity

Models	Control	Case OR (95% CI)	*P*
Model 1	1	1.02 (0.92, 1.13)	0.68
Model 2	1	1.03 (0.93, 1.15)	0.56
Model 3	1	1.06 (0.94,1.20)	0.33

^#^Results were calculated using conditional logistic regression analyses

Model 1 is conditioned on the matching factors of SBP and DBP

Model 2 is conditioned on the matching factors of SBP and DBP, and adjusted for energy intake and smoking

Model 3 is the Model 2 plus further adjusted for hypertension, diabetes, hyperlipidemia, and overweight

### Discussion

This nested case-control study aimed to investigate the relationship between DTAC and the risk of stroke. The results of this study did not show a significant association between DTAC and stroke risk, even after adjusting for potential confounding factors such as age, sex, event time, energy intake, smoking, hypertension, and diabetes.

Numerous research studies have explored the role of dietary factors in the development of stroke. However, a limited amount of information is available regarding the connection between DTAC and stroke. In line with our findings, in a large population-based cohort study among Swedish women, it has been reported a non-significant correlation between DTAC and stroke risk [34]. In addition, based on the results of another cohort study among Italian adults, it has been shown that there wasn’t any significant correlation between DTAC and hazard ratio (HR) for all types of strokes. However, they found a significant inverse correlation between DTAC and the risk of ischemic stroke [33]. Contrary to our findings, the results of some other studies show the protective effect of DTAC against the risk of stroke. Milajerdi et al., in a case–control study, showed an inverse relationship between DTAC and the risk of stroke [22]. Similar findings were reported in another study [20, 45, 46].

The lack of a significant association between DTAC and stroke risk in this study may be due to several reasons. One of the primary reasons is the variation in individuals’ eating habits and lifestyles among different countries [47]. Furthermore, the employment of distinct methods for measuring DTAC, along with differences in the types of dietary questionnaires employed, are additional factors that contribute to this discrepancy and impact the precision of the findings. Another reason for the contradiction observed in the results of the studies is the difference in the patients’ age range and demographic characteristics. Contrary to our findings Yang et al., in their study and the subgroup analysis, revealed that individuals over 60 years old, women, drinkers, former smokers, and those with no physical activity exhibited a stronger correlation between lower DTAC and an elevated risk of stroke [20].

Our study boasts several strengths. Firstly, we utilized DTAC to comprehensively measure the overall antioxidant potential derived from dietary sources. In addition, we carefully adjusted for potential confounding factors, particularly considering the contentious nature of the association between DTAC and stroke, which incorporates lifestyle and health-related variables.

However, our study also had some limitations that should be noted. Firstly, DTAC is a complex measure that takes into account the antioxidant capacity of multiple nutrients and phytochemicals in the diet. It is possible that the individual contribution of each antioxidant nutrient to stroke risk reduction is small, and the combined effect of all antioxidants may not be significant. Secondly, the measurement of DTAC is subject to measurement error, as it relies on the accuracy of dietary assessment methods and the FRAP database. Furthermore, our study’s generalizability may be constrained by its geographical and demographic scope. Focused solely on a rural area in Iran, our findings may not be fully representative of broader populations with differing dietary habits, lifestyles, and stroke risk profiles. Additionally, the relatively modest sample size of our study may have limited statistical power, potentially hindering our ability to detect significant associations between DTAC and stroke risk. Additionally, as a nested case-control study, our research design inherently possesses certain limitations. While nested case-control studies offer advantages such as efficient data collection and reduced costs, they are susceptible to biases related to the selection of controls from within the cohort. The potential for selection bias or misclassification of exposure variables must be acknowledged, as these factors could influence the validity and interpretation of our results.

Moreover, the reliance on questionnaire-based data collection introduces inherent limitations related to recall bias and respondent accuracy. Despite efforts to mitigate these biases through rigorous questionnaire design and validation procedures, the possibility of inaccuracies in reported dietary intake and lifestyle factors cannot be entirely eliminated.

## Conclusion

Our study suggests that the measured DTAC may not be a significant predictor of stroke risk in this particular population. Although we observed dietary patterns, and antioxidant intake differences between cases and controls and among DTAC tertiles, the multivariate-adjusted analysis did not reveal any significant association between DTAC and stroke risk. These findings underscore the complex interplay of factors that influence stroke risk and highlight the need for further research to comprehensively understand these relationships.

## Data Availability

The datasets used and/or analysed during the current study are available from the corresponding author on reasonable request.
